# ATF3 Protects against LPS-Induced Inflammation in Mice via Inhibiting HMGB1 Expression

**DOI:** 10.1155/2013/716481

**Published:** 2013-08-26

**Authors:** Pei-Fang Lai, Ching-Feng Cheng, Heng Lin, Tzu-Ling Tseng, Hsi-Hsien Chen, Sung-Ho Chen

**Affiliations:** ^1^PhD Program in Pharmacology and Toxicology, Tzu-Chi University, No. 701, Chung Yang Road, Section 3, Hualien 97004, Taiwan; ^2^Department of Emergency Medicine, Buddhist Tzu Chi General Hospital, No. 707, Section 3, Chung Yang Road, Hualien 97004, Taiwan; ^3^Department of Medical Research, Buddhist Tzu Chi General Hospital and Department of Pediatrics, Tzu Chi University, No. 707, Section 3, Chung Yang Road, Hualien 97004, Taiwan; ^4^Department of Physiology, School of Medicine, College of Medicine, Taipei Medical University, 250 Wu-Hsing Street, Taipei City 110, Taiwan; ^5^Institutes of Medical Sciences, College of Medicine, Tzu-Chi University, No. 701, Chung Yang Road, Section 3, Hualien 97004, Taiwan; ^6^Center of Vascular Medicine, College of Life Sciences, Tzu-Chi University, No. 701, Chung Yang Road, Section 3, Hualien 97004, Taiwan; ^7^Division of Nephrology, Department of Internal Medicine, Taipei Medical University Hospital, No. 252, Wu Hsing Street, Taipei City 110, Taiwan; ^8^Department of Internal Medicine, School of Medicine, College of Medicine, Taipei Medical University, 250 Wu-Hsing Street, Taipei City 110, Taiwan; ^9^Department of pharmacology, Tzu-Chi University, No. 701, Chung Yang Road, Section 3, Hualien 97004, Taiwan

## Abstract

Lipopolysaccharide (LPS) triggers innate immunity mainly via TLR4 signaling. ATF3 is a negative regulator of TLR4 signaling. HMGB1 plays a critical role in the final step of sepsis. However, the mechanisms of ATF3 and the role of HMGB1 in regulating innate immunity-induced sepsis are incompletely understood. In this study, we found that serum HMGB1 levels were 10-fold higher in patients with sepsis than normal controls. We further demonstrated that ATF3 gene knockout in mice subjected to LPS-induced endotoxemia correlates with an increase in the mortality rate and the elevated expression of IL-6, TNF-**α**, NO, MCP-1, and HMGB1 in the lung tissues or serum. The biochemical effects of ATF3 were observed in *in vitro* macrophages and blocked by ATF3 siRNA treatment. We have also shown that adeno-associated virus-mediated ATF3 gene transfer protected ATF3 knockout mice from LPS-induced mortality. In addition, ATF3 knockdown increased LPS-induced release of HMGB1. In conclusion, upregulation of ATF3 contributes to the reduced release of inflammatory molecules, especially HMGB1, which induced lung injury and increased the survival rate of mice after LPS challenge. Therefore, suppressing LPS-induced inflammation with ATF3 induction or ATF3 mimetics may be an important strategy for sepsis therapy.

## 1. Introduction

Sepsis, a major cause of morbidity and mortality worldwide [1], occurs in 18 to 42% of patients with Gram-negative bacterial infection [2]. Lipopolysaccharides (LPS) are endotoxins derived from the outer membranes of Gram-negative bacteria and are main triggers of innate immunity and acute inflammation that are vital for antimicrobial defense reactions [3]. LPS binds to Toll-like receptor 4 (TLR4) to activate a crucial proinflammatory transcription factor NF-*κ*B that elicits expressions of several proinflammatory cytokines and chemokines such as TNF-*α*, IL-6 [4], and MCP-1 [5].

Analyses of endotoxin-stimulated macrophages revealed that ATF3 is a rapidly induced transcription factor that represses IL-6 transcription [6, 7]. ATF3, a member of the ATF/cyclic adenosine monophosphate (cAMP) responsive element-binding protein (ATF/CREB) family of transcription factors, is a stress-inducible transcription factor. The ATF3 promoter comprises several TLR-responsive elements such as AP-1 and NF-*κ*B sites; hence, the early phase of ATF3 induction is a foremost response to TLR signaling [8]. In addition, ATF3 induction during sepsis augments susceptibility to secondary infections [9]. ATF3 has been implicated as a strong target for prophylactic or therapeutic agent in endotoxic shock or bacterial sepsis [9].

High mobility group box 1 (HMGB1), a damage-associated molecular pattern (DAMP) [10], serves as a transcription factor in the nucleus and also as a proinflammatory cytokine when released into the extracellular fluids [11, 12]. HMGB1 is involved in the activation of innate immune mechanisms in the context of viral and bacterial infections [13]. Clinical reports reveal that its levels are increased significantly in critically ill patients with sepsis [14, 15]. Protective effects of activated protein C in severe sepsis may partially be mediated through the inhibition of HMGB1 signaling [16]. Receptor for advanced glycation end products (RAGE) and certain TLRs serve as the major signaling receptors for HMGB1 at the cell surface and inside the cell. Further, HMGB1 interacts specifically with TLR4/MD2 and activates macrophages and monocytes to release proinflammatory cytokines such as TNF*α* [17, 18].

The role of ATF3 and HMGB1 in immune regulation and their connections to inflammatory diseases have been reported, and they both function in TLR-related pathways. However, it is unclear whether ATF3 plays a role in the HMGB1 regulation of LPS-induced endotoxemia. The purpose of this study is to characterize the relation between ATF3 and HMGB1. We found that ATF3 protects against LPS-induced endotoxemia in mice through reducing HMGB1 expression.

## 2. Methods and Materials

### 2.1. Animal Source

The ATF3-KO mice were kindly provided by Dr. Tsonwin Hai (Ohio State University, Columbus, USA). The ATF3-KO mice allele was backcrossed into C57BL/6J (B6) mice for at least seven generations before LPS-induced endotoxemic experiments. Male mice (8 to 10 weeks old and weighing 25–35 g) maintained under standard conditions at Tzu Chi University's Animal Center were used. All experimental procedures were approved by the Animal Care and Use Committee of Tzu Chi University.

### 2.2. Experimental Procedures

LPS serotype 0127:B8 (Sigma-Aldrich Chemical, St. Louis, USA) was dissolved in sterile physiological saline immediately before use. Mice were divided randomly into 4 groups, administered LPS (5 or 50 mg/kg, ip), and sacrificed at 0, 3, 6, and 24 hrs after LPS challenge under anesthesia (pentobarbital, 50 mg/kg, ip), and the lung tissues were removed for subsequent experiments. Supernatants of blood samples also were collected and frozen at −80°C for subsequent assays. In another study, mice were injected intraperitoneally with recombinant AAV (adeno-associated virus)-PGK (phosphoglycerate kinase) or AAV-ATF3 vectors (2 × 10^8^ viruses). Two weeks later, mice were treated with LPS (5 mg/kg, ip) for 24 hrs, and the lungs were removed for further studies.

### 2.3. Culture of RAW 264.7 Macrophage Cell Line

RAW 264.7 cells were obtained from the Bioresource Collection and Research Center (Hsinchu, Taiwan). Cells were cultured in a high-glucose DMEM medium containing 10% heat-inactivated fetal bovine serum (FBS, Hyclone Laboratories, Logan, USA), antibiotics (100 U/mL penicillin, 0.1 mg/mL streptomycin, and 0.25 **μ**g/mL amphotericin, Biological Industries, Beit Haemek, Israel) and maintained at 37°C in a humidified incubator containing 5% CO_2_. Cells were cotransfected with plasmids using lipofectamine 2000 (Invitrogen, Carlsbad, USA). Three days after transfection, cells were treated with LPS (200 ng/mL) for 24 hrs.

### 2.4. Plasma TNF-*α* and IL-6 Measurement

Plasma TNF-*α*, IL-6, and HMGB1 concentrations were measured by enzyme-linked immunosorbent assay (TNF-*α* and IL-6: from Enzo Life Science, USA; HMGB1: from Uscn Life Science, Houston, USA) according to manufacturer's instructions.

### 2.5. IL-6 and TNF-*α* mRNA Measurement

IL-6 and TNF-*α* mRNA were measured by RT-PCR using a Super Script kit (Invitrogen) according to manufacturer's instructions. Total RNA was extracted from the lung tissue using trizol (Invitrogen) according to our previous report [19]. mRNA samples were quantified by spectrophotometer, and equal amounts of mRNA were reversely transcribed into first-strand cDNA. PCR amplifications were performed in triplicate using mixture of Master SYBR Green supermix (Roche, Switzerland). cDNA and specific primers for TNF-*α* and IL-6 were listed in Table 1. The real-time PCR was performed for 45 cycles of 95°C for 15 s and 60°C for one minute using an ABI Prism 7300 (Life Technologies, USA).

### 2.6. Plasma and Medium NO Measurement

Plasma and medium NO were measured by conventional Griess reaction. Standard procedures were used for the preparation of plasma and medium samples for NO assay. Plasma and medium NO concentrations were calculated from the standard nitrite curve.

### 2.7. Immunofluorescence (IF)

Standard IF techniques were used. Briefly, 2 *μ*m paraffin-embedded tissue sections from the lungs were processed through antigen retrieval, permeabilized with Triton X-100, blocking nonspecific interaction with normal serum (Biogenx, Fremont, USA), followed by washing and reaction with goat MCP-1 polyclonal antibody (1 : 50, Santa Cruz Biotechnology, Santa Cruz, USA) and mouse HMGB1 monoclonal antibody (1 : 100, ABCAM, Cambridge, UK). The secondary antibodies were rhodamine red-linked anti-mouse IgG (1 : 200, KPL,Washington, USA) and FITC-conjugated anti-goat IgG antibody (1 : 200, KPL). Cell nuclei were counter-stained with DAPI (1 : 200, KPL). All sections were mounted with a water-soluble mounting medium and examined under a fluorescence microscope (Leica, Leica Microsystems,Wetzlar, Germany).

### 2.8. Isolation of Nuclear and Cytosolic HMGB1 and NF-*κ*B Proteins

Frozen lung tissues were homogenized in a buffer containing PBS and phosphatase inhibitors (Nuclear extract kit, Active Motif, Carlsbad, USA) and centrifuged at 500 rpm for 5 min. The resulting pellets were resuspended in 500 *μ*L hypotonic buffer and incubated for 15 min. Nuclei were isolated by centrifugation at 10,000 rpm for 1 min. The supernatants containing cytoplasmic and membrane proteins were collected and stored at −80°C. The pellets were resuspended in 50 *μ*L complete lysis buffer and centrifuged at 10,000 rpm for 10 min. The supernatants containing the nuclear extracts were collected and stored at −80°C for immunoblotting.

### 2.9. Western Blotting

Protein concentrations were quantified by bicinchoninic acid (BCA) protein assay reagent (Pierce Chemicals, Rockford, USA). Standard techniques for SDS polyacrylamide gel electrophoresis and immunoblotting were followed. The antibodies used were mouse iNOS monoclonal IgG (1 : 500, BD Pharmagen, California, USA), mouse HMGB1 monoclonal IgG (1 : 1000, Abcam), rabbit ATF3 polyclonal IgG (1 : 1000, Santa Cruz Biotechnology), rabbit NF-*κ*B monoclonal antibodies (1 : 500, Santa Cruz Biotechnology), mouse antiactin antibody (1 : 4000, Chemicon, IL, USA), and rabbit histone H2A polyclonal antibody (1 : 2000, Cell Signaling Technology, Beverly, CA, USA). The immunoreactivities were visualized and scanned into a computer. Individual bands were analyzed by Image J software (National Institute of Mental Health, NIH, Bethesda, USA).

### 2.10. Histopathology

Standard protocols were followed [20]. Briefly, lung specimens were fixed in 4% paraformaldehyde, dehydrated through a graded series of ethanol, and embedded in paraffin, sectioned at 2 *μ*m. Sections on slides were deparaffinized in nonxylene solution, rehydrated, and stained with hematoxylin and eosin. The specimens were examined under a light microscope (Leica Microsystems).

### 2.11. Lung Injury Assessment

The lung injury scores were estimated based on histological examination, including the hemorrhage of intraalveolar septa, cell infiltration, and lung wet-to-dry ratio. The scores were determined as 0 (no injury), 1 (10–20% severity), 2 (20–40% severity), 3 (40–50% severity), or 4 (greater than 50% severity) [21]. The degree of intra-alveolar septa hemorrhage was scored as 0 (no hemorrhage), 1 (mild hemorrhage), or 2 (severe hemorrhage). The thickness of intraalveolar septa was quantified by measuring all septae along a crosshair placed on each image (20 septae for each animal) using Image J software. Lung wet-to-dry ratio that determines pulmonary edema was scored as 0 (no edema), 1 (mild edema), or 2 (severe edema) as compared with the control group.

### 2.12. Measurement of the Survival Rate in Mice

C57BL/6J (B6) and ATF3-KO mice were used to evaluate the survival rate [22]. Mice were given lethal dose of LPS (50 mg/kg, ip) to induce endotoxemia and were divided randomly into several groups and examined at different intervals for 24 hrs.

### 2.13. Adeno-Associated Virus Preparation and Gene Transfer

The procedure was similar to those described previously [23]. We constructed in the replication-defective recombinant adenoviral vector a human phosphoglycerate kinase (PGK) promoter to drive ATF3 expression (AAV-ATF3) and a GFP promoter alone to serve as a control (AAV-GFP). Replication-defective recombinant adenoviral vectors were amplified and titrated in HEK-293 cells as described previously [22]. For adenovirus-mediated gene transfer, cells were exposed to adenoviral vectors at the indicated multiplicity of infection.

### 2.14. Statistical Analysis

The experimental data were presented as means ± SEM. The one-way and two-way ANOVA were used to determine the difference. Measurements at single time point were compared by the Student's unpaired *t*-test. A *P* value of less than 0.05 was considered statistically significant. The survival rate was analyzed by Kaplan-Meyer survival curves.

## 3. Results

### 3.1. ATF3 Expression Was Upregulated in Mice after LPS Injection

**Figure 1:**
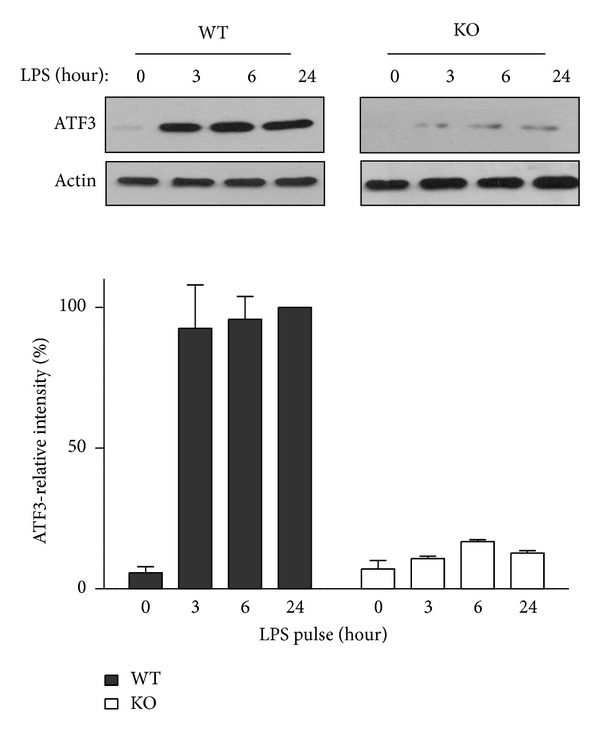
Expression level of ATF3 in the lung tissues of WT (wild type) and KO (ATF3 knockout) mice challenged with LPS. Representative immunoblots of ATF3 protein in the lung tissue lysates from WT and KO mice at 0, 3, 6, and 24 hrs after LPS administration (5 mg/kg, ip) were shown. The intensity of ATF3 protein was normalized to actin which was served as the internal control. The relative intensity at 24 hrs from WT mice was set at 100%. Data represent mean ± SEM. *n* = 8 in each group.

The level of ATF3 in the lung tissues of a mouse sepsis model was measured to identify the role of ATF3 in LPS-induced sepsis. Immunoblot analysis revealed increased level of ATF3 protein in the wild-type (WT) animals as early as 3 hrs after LPS (5 mg/kg, ip) treatment and maintained at the elevated level for up to 24 hrs. However, low level of ATF3 was detected in the lung tissues from the ATF3-knockout (KO) mice (Figure 1).

### 3.2. The Survival Rate of Mice under LPS-Induced Endotoxemia Was Reduced after ATF3 Knockout

**Figure 2:**
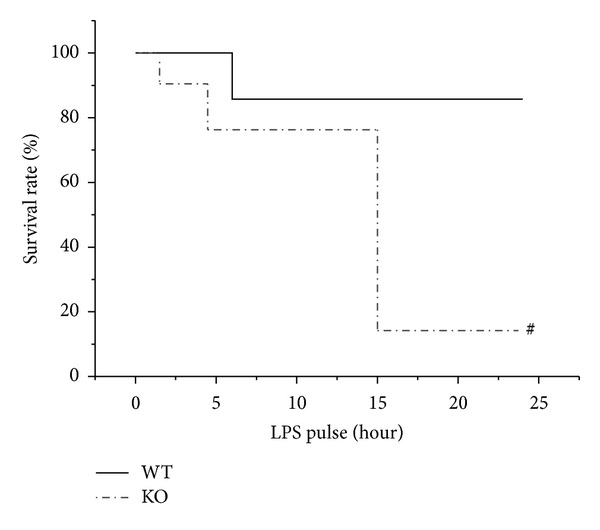
The Kaplan-Meyer survival rate of WT (wild type) and KO (ATF3 knockout) mice at 0, 3, 6, and 24 hrs following administration of a lethal dosage of LPS (50 mg/kg, ip). Data represent three independent experiments (21–24 animals used). #, *P* < 0.05, WT versus KO mice.

Sepsis and septic shock are thorny problems, which cause high mortality from severe inflammatory response resulting in multiple organs dysfunction [24]. To confirm the pathological involvement of ATF3 in LPS-induced endotoxemia, WT and ATF3-KO mice were administered a lethal dose of LPS (50 mg/kg, ip), and the survival rate was examined.  Figure 2 shows that less than 20% of the LPS-treated ATF3-KO mice survived for 24 hrs. In contrast, greater than 80% of WT mice survived after 24 hrs. These observations suggested that enhanced expression of ATF3 may protect mice from LPS-induced shock.

### 3.3. ATF3 Knockout Increased MCP-1 Expression and Inflammation-Induced Lung Injury in Mice Induced by LPS

**Figure 3:**
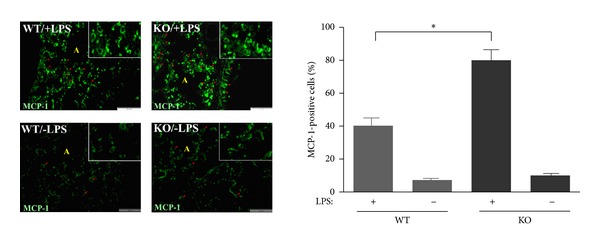
ATF3 knockout increased LPS-induced MCP-1 level in the lung tissues of mice. Localization of MCP-1 induced by LPS in the lung tissues of WT and ATF3-KO mice detected by immunofluorescence. A: alveoli. Red arrows show MCP-1 staining of alveolar septa with few neutrophils. Insets show MCP-1 expression in the cytoplasm. Scale bar = 50 *μ*m. MCP-1-positive cells were calculated from 4 fields with a total number of 200 cells counted. Data represent mean ± SEM (*n* = 4). ∗, *P* < 0.01.

**Figure 4:**
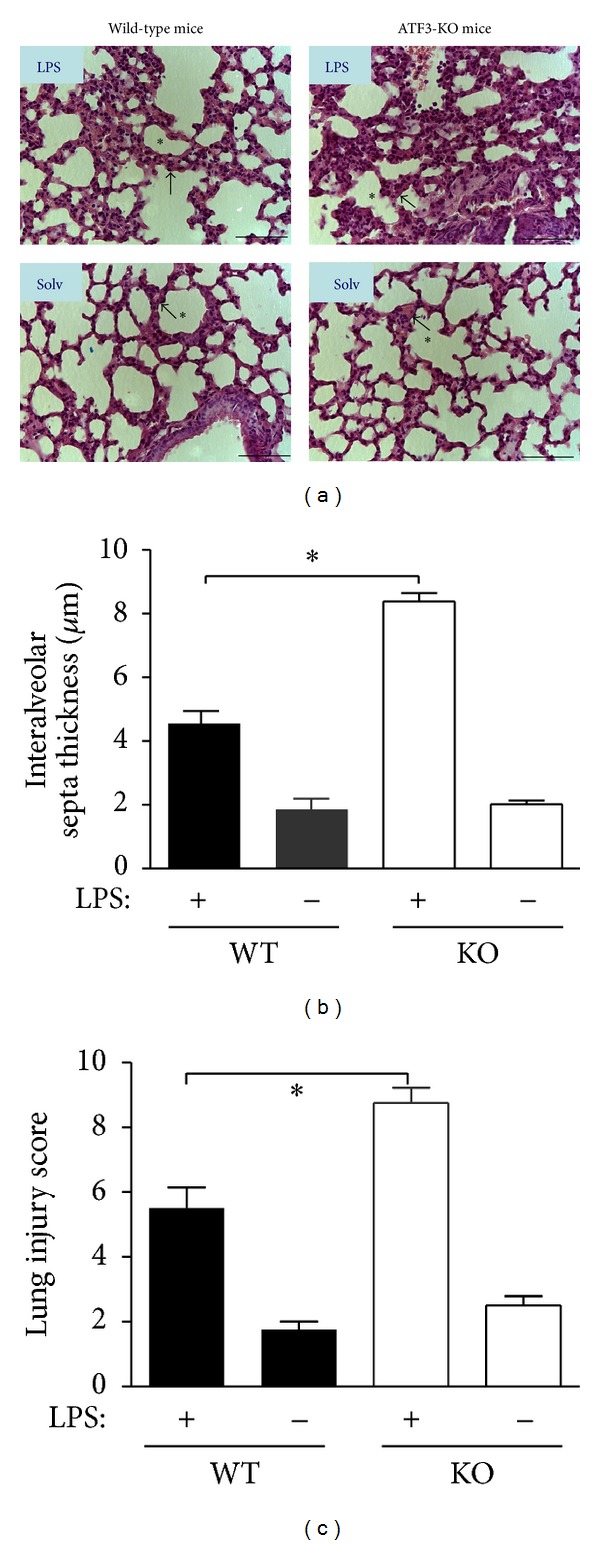
ATF3 inhibited polymorphonuclear leukocyte infiltration and lung injury in mice after LPS treatment. (a) Representative sections of the lung tissues were stained with hematoxylin and eosin. Few neutrophils were present in control WT sections. Solv, normal saline;* asterisk*, normal alveoli; *arrow*, alveolar septa. Thickened septa were observed 24 hrs after LPS (5 mg/kg, ip) treatment. Scale bar, 20 *μ*m. Intra-alveolar septal thickness (b) and lung injury score (c) were significantly increased 24 hrs after LPS challenge (5 mg/kg, ip) in ATF3-KO mice compared to WT mice. Data represent mean ± SEM (*n* = 4). ∗, *P* < 0.01.

It has been shown that the secretion of chemokine, MCP-1, by LPS-activated endothelial cells contributes substantially to the pathogenesis of sepsis [25]. However, the mechanism involved in LPS-induced MCP-1 production in the lung is not well understood. We examined the expression of MCP-1 in the lungs of WT and ATF3^−/−^ mice under LPS treatment. As shown in Figure 3, ATF3^−/−^ mice showed marked elevated level of MCP-1 compared with WT controls (Figure 3(a)), and MCP-1 predominately was expressed within the cytoplasmic region of MCP-1-positive cells (Figure 3(a) and insets). Furthermore, we compared the lung injury score in mice after LPS injection, and histological analysis of the lung tissues from LPS-treated mice showed that ATF3 knockout further increased the accumulation of activated alveolar macrophages (Figure 4(a)) and the thickness of intra-alveolar septa (Figure 4(b)). Similarly, ATF3^−/−^  mice revealed augmented lung injury score caused by LPS (Figure 4(c)). Together, these results indicate that ATF3 can reduce MCP-1 expression and decrease lung injury after sepsis occurrence in mice.

**Figure 5:**
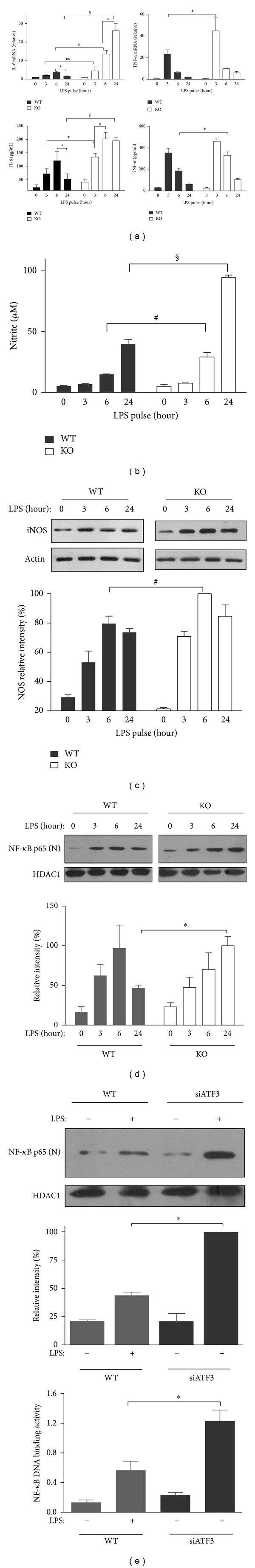
Effects of ATF3 knockout on LPS-induced expression of IL-6, TNF-*α*, and iNOS and translocation of NF-*κ*B p65 in mice and in macrophages. (a) Quantitative RT-PCR (mRNA, top panels) and ELISAs (protein, lower panels) of Il-6 and TNF-*α* from the lung tissues of WT and ATF3^−/−^ mice treated with LPS (5 mg/kg, ip). Expression of mRNA was normalized to that of glyceraldehyde 3-phosphate dehydrogenase. Data represent mean ± SEM (*n* = 4). ∗, *※*, #, and §, *P* < 0.05; NS indicates not significant. (b) shows plasma nitrite concentrations. Data represent mean ± SEM (*n* = 6). #, *P* < 0.01. (c) shows a representative Western blots of iNOS. The intensity of iNOS was normalized to that of *β*-actin which was served as the loading controls. Data represent mean ± SEM (*n* = 3). #, *P* < 0.01. (d) Representative Western blots of nuclear NF-*κ*B p65 protein in the lung tissues of WT and KO mice after LPS treatment. HDAC1 was served as the internal standard. Data represent mean ± SEM (*n* = *x*). ∗, *P* < 0.01. (e) Nuclear NF-*κ*B p65 levels in RAW264.7 macrophages measured by Western blot analysis. The intensity of nuclear p65 was normalized to that of HDAC1. Nuclear NF-*κ*B DNA binding activity in RAW264.7 macrophages measured by ELISAs in RAW264.7 macrophages after incubation with LPS (200 ng/mL) for 24 hrs. Data represent mean ± SEM (*n* = 3). ∗, *P* < 0.01.

Recent studies have indicated the involvement of NF-*κ*B in MCP-1 induced IL-6 activation in epithelial cell or in allergic airways disease [26, 27]. Therefore, we hypothesized that ATF3 may modulate the expression of IL-6 and TNF-*α* in the early and later stages of endotoxemia.  Figure 5(a) shows that the mRNA and protein level of TNF-*α* or IL-6 in the lung tissues or in the serum were significantly higher at 3 or 6 hrs, respectively, in ATF3^−/−^ mice compared to WT mice following LPS administration.

It has been reported that MCP-1^(−/−)^ mice showed reduced macrophage infiltration and expression of tumor-necrosis factor-alpha (TNF)-*α* and iNOS [28]. Therefore, we examined the plasma nitrite concentration, a derived metabolite of NO, and iNOS expression in the lung tissues of WT and ATF3^−/−^ mice. ATF3 knockout increased the LPS-induced plasma nitrite elevation, which was continuously increased up to 24 hrs examined. The concentration of plasma nitrite at 24 hrs in ATF3-KO mice was about 2-fold of that in WT mice (Figure 5(b)), and iNOS protein expression level was significantly increased in ATF3^−/−^ mice compared to WT mice after LPS treatment (Figure 5(c)).

Following LPS treatment, NF-*κ*B is translocated into the nucleus to drive the expression of various inflammatory genes, which are implicated in the pathogenesis of sepsis [25]. Because IL-6, TNF*α*, and MCP-1 gene promoters contain functional NF-*κ*B binding sites essential for their induction in response to inflammatory stimuli, we further explored NF-*κ*B activation by Western blotting. The activation and translocation of NF-*κ*B were markedly increased in ATF3^−/−^ mice compared to WT mice 24 hrs after LPS treatment (Figure 5(d)). We also used the loss of function assay to inhibit ATF3 function in macrophages (RAW264.7). As shown in Figure 5(e), NF-*κ*B nuclear translocation (left panel) and NF-*κ*B binding activity (right panel) both were significantly higher in the cells receiving ATF3 siRNA transfection. These results suggest that reduction of ATF3 upregulates the expression of inflammatory cytokine or NO through an NF-*κ*B-dependent pathway after LPS injury in the lung.

### 3.4. Patients with Sepsis Syndrome Had Elevated Serum HMGB1 Levels

**Figure 6:**
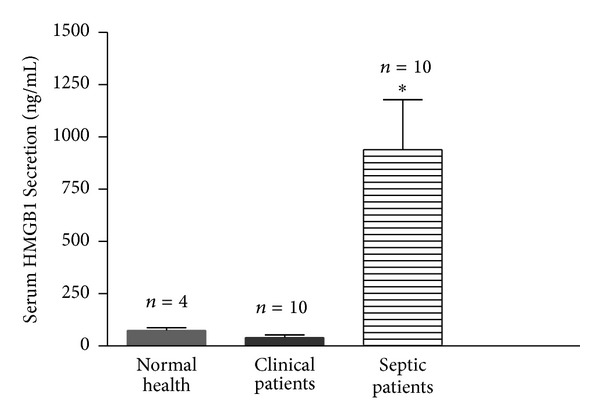
Serum HMGB1 levels in septic patients. Serum HMGB1 was measured by an ELISA kit in the healthy volunteers and inpatients with or without sepsis. ∗, *P* < 0.05.

Both ATF3 and MCP-1 have been reported to be novel biomarkers for detecting inflammation-induced nephropathology [29, 30]. Since ATF3 modulates the secretion and release of HMGB1, which is a down-stream proinflammatory mediator and may more closely reflect the status of sepsis, we, therefore, examined its level in sepsis patients. In a preliminary human study, serum samples were collected from ten inpatients whose diagnosis indicated sepsis, ten inpatients without infection, and four healthy volunteers. The demographic profile of these subjects is listed in Table 2. The serum levels of HMGB1 of inpatients suffering from sepsis were significantly higher when compared to the control groups (Figure 6). These results indicate that elevated serum HMGB1 level reflects the severity of sepsis syndrome.

### 3.5. *In Vivo *ATF3 Gene Transfer Reversed LPS-Induced Lethality in ATF3^−/−^ Mice

**Figure 7:**
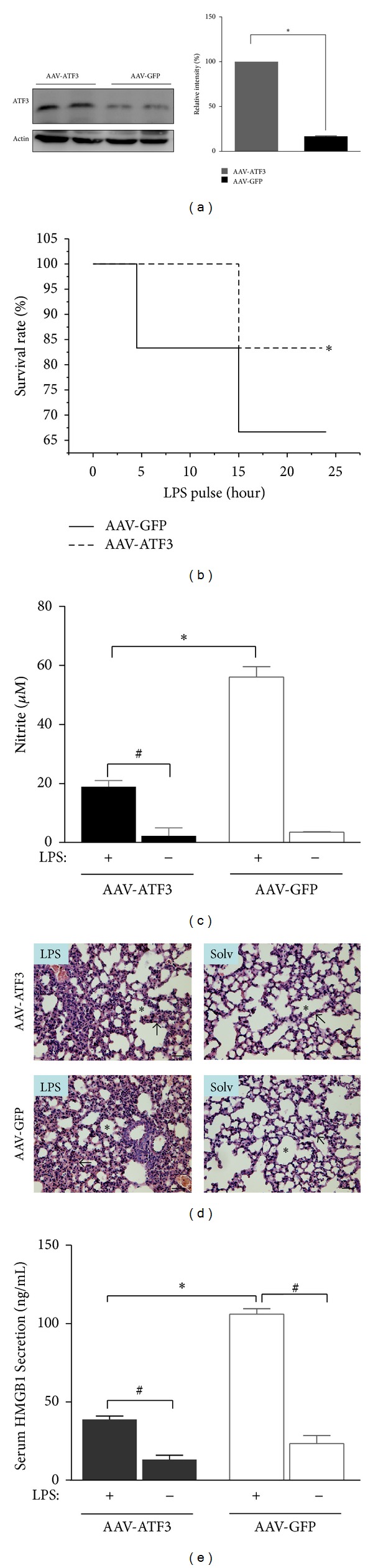
Reintroduction of ATF3 by adeno-associated virus increased the survival rate, attenuated lung injury, and reduced serum nitrite and HBGB1 concentrations in ATF3 KO mice following LPS treatment. (a) Representative Western blots of adenovirus- (AAV-) mediated ATF3 expression in ATF3^−/−^ mice. AAV-GFP (phosphoglycerate kinase) was used as the control vector. Data represent mean ± SEM (*n* = 16). ∗, *P* < 0.01. (b) Kaplan-Meyer survival rate of mice following treatment with LPS (50 mg/kg, ip). Data represent mean ± SEM (total 21–24). ∗, *P* < 0.01. (c) Plasma nitrite levels were measured at 24 hrs after LPS (5 mg/kg, ip) challenge. Data represent mean ± SEM (*n* = 3). ∗, #, *P* < 0.01. (d) Representative sections of the lung tissues were stained with hematoxylin and eosin. In control sections (solv denotes normal saline, without LPS challenge), normal alveoli (asterisk) and alveolar septa (arrow) with few neutrophils were observed. Scale bar, 20 *μ*m. (e) Serum HMGB1 concentrations at 24 hrs after LPS challenge analyzed by ELISAs. Data represent mean ± SEM (*n* = 3). ∗, #, *P* < 0.01. Dosages of AAV, 2 × 10^8^ virus.

The preceding observations suggest that restoration of ATF3 expression in ATF3 knockout mice would be expected to reduce LPS-induced sepsis injury in these mice. And as a complementary approach to the knockout mouse studies, AAV8, which provides high-level and stable gene expression in the mouse and rat heart, was explored. Delivery of AAV8-ATF3 gene to ATF3^−/−^ mice restored ATF3 protein in the lung tissues (Figure 7(a)). LPS-induced lethality in ATF3 knockout mice was reversed by ATF3 gene transfer as reflected by increased survival rate (Figure 7(b)). Similarly, elevated plasma nitrite and serum HMGB1 levels and lung injury caused by LPS in ATF3 knockout mice were also reversed by ATF3 gene transfer (Figures 7(c)–7(e)).

### 3.6. ATF3 Modulated HMGB1 Release in LPS-Induced Endotoxemia in Mice

**Figure 8:**
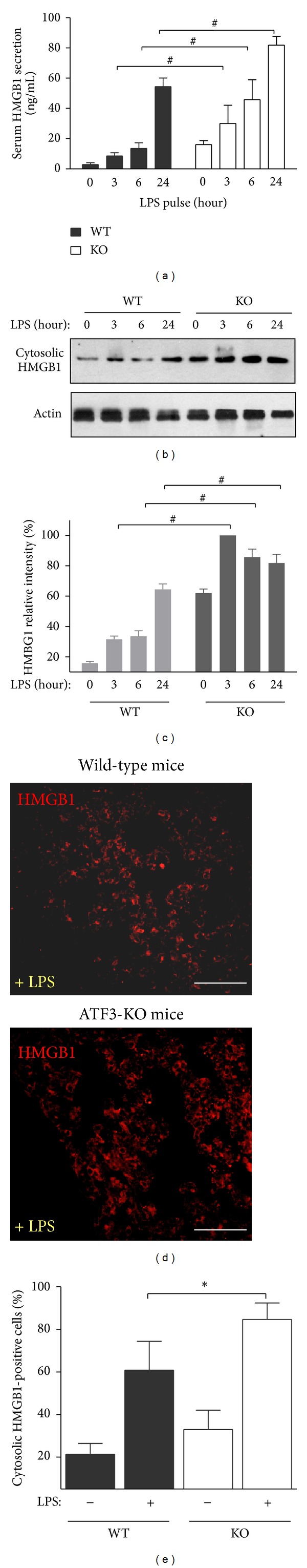
ATF3 knockout enhanced LPS-induced increase in serum and cytosolic HMGB1 concentrations in mice. (a) Serum HMGB1 concentrations after LPS challenge (5 mg/kg, ip) analyzed by ELISAs. Data represent mean ± SEM (*n* = 3). #, *P* < 0.01. (b) Representative Western blots of cytosolic HMGB1 expression in the lung tissues. The relative percentage of cytosolic HMGB1 protein normalized to actin, which was served as the internal control, was shown in (c). Data represent mean ± SEM (*n* = 3). #, *P* < 0.01. (d) Immunofluorescent localization of cytosolic HMGB-1 induced by LPS in the lung tissues of wild type and ATF3-KO mice. Scale bar, 50 *μ*m. (e) The percentage of cells expressing cytosolic HMGB1-1 was calculated from 4 fields with a total number of 200 cells examined. Data represent mean ± SEM (*n* = 4). ∗, *P* < 0.01.

**Figure 9:**
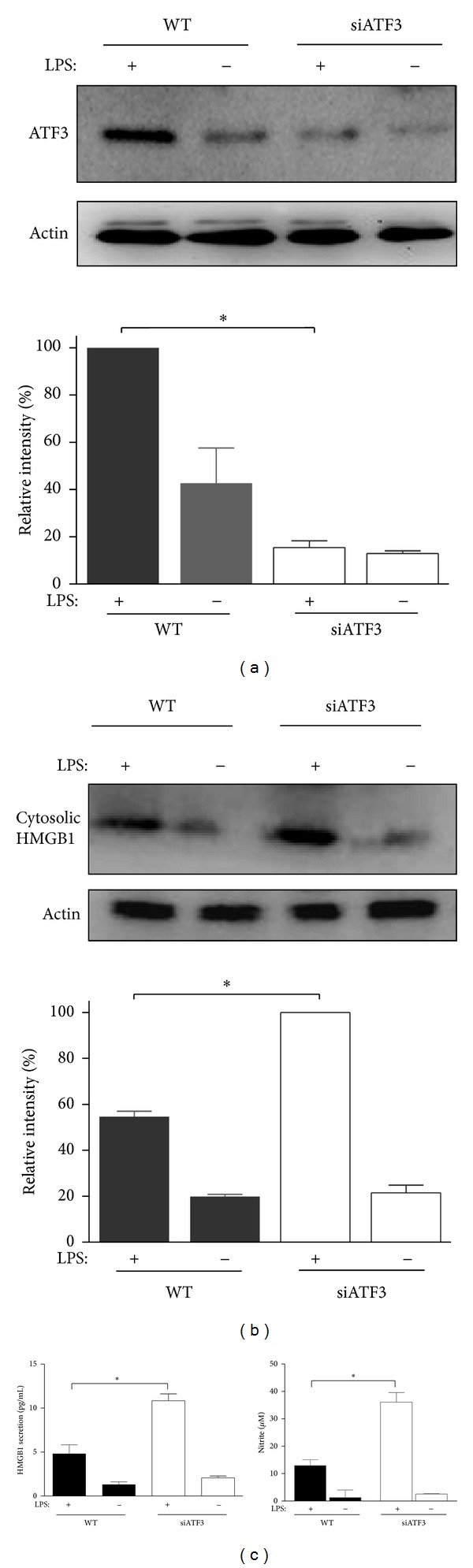
ATF3 siRNA enhanced LPS-induced elevation of ATF3 and cytosolic HMGB1 expression and medium HMGB1 and nitrite concentrations in macrophage culture. (a) Representative Western blots of ATF3 expression in RAW264.7 cells treated with LPS (200 ng/mL) and ATF3 siRNA. The intensity of ATF3 was normalized to that of actin, which was served as the internal standard, and the percentage of control in the presence of LPS (200 ng/mL) was set as 100%. Data represent mean ± SEM (*n* = 3). ∗, *P* < 0.01. (b) Western blots of cytosolic HMGB1 expression. The intensity of cytosoloic HMGB1 was normalized to that of actin, and the relative intensity in the presence of LPS + siATF3 was taken as 100%. Data represent mean ± SEM (*n* = 3). ∗, *P* < 0.01. (c) HMGB1 and nitrite levels in the medium measured by ELISAs after treatment of macrophages with LPS. Data represent mean ± SEM (*n* = 4). ∗, *P* < 0.01.

Recent studies have shown that administration of a neutralizing antibody to *HMGB1* significantly lowered the levels of IL-6, TNF*α*, and MCP1 mRNA. In addition, TLR4 deficient mice were protected against ischemia-reperfusion-induced kidney injury; administration of neither anti-HMGB1 antibody nor rHMGB1 affected this renoprotection [31]. These data suggest that ATF3 may be involved in the regulation of HMGB1. We found that the concentration of circulating HMGB1 was relatively low in WT mice, but its level was increased by about 4-5-folds 3 to 24 hrs following LPS challenge (Figure 8(a)). Similarly, a sustained and higher level of cytoplasmic HMGB1 release was observed 3 to 24 hrs after LPS challenge (Figures 8(b) and 8(c)). In addition, LPS elicited the release of nuclear HMGB1 into the cytoplasm of numerous cells in the lung tissues (Figure 8(d)), and the percentage of HMGB1-positive cells was significantly increased in ATF3^−/−^ compared to WT mice following LPS administration (Figure 8(e)). We further used the loss of function assay to inhibit ATF3 function in *in vitro* system. Cultured macrophages (RAW264.7) were transfected with ATF3 siRNA and control vector plasmid followed by LPS treatment. As shown in Figure 9, LPS-induced ATF3 gene expression was blocked as indicated by Western blotting (Figure 9(a)). HMGB1 release and secretion (Figure 9(b)), and NO (Figure 9(c)) induced by LPS were markedly enhanced when ATF3 functions were silenced by ATF3 siRNA compared with control cells.

## 4. Discussion

Although ATF3 expression is believed to play an important role in response to various stresses [8], little is known about its function in endotoxemia. In this study, we demonstrated that ATF3 exhibited a protective effect against LPS- induced endotoxemia in mice. ATF3 expression not only decreased LPS-induced elevation inflammatory mediators, such as IL-6, TNF-*α*, NO, iNOS, MCP-1, and HMGB1, in the lung tissues and blood, but also increased the survival rate in endotoxemic mice. The upstream regulator of these inflammatory mediators is TLR4-NF-*κ*B pathway [7], and ATF3 negatively regulates this pathway. We showed that *atf3 *deficiency in mice could augment NF-*κ*B presentation, and treatment of macrophages with ATF3 siRNA enhanced the nuclear translocation of NF-*κ*B p65 (Figures 5(d) and 5(e)). We also found elevated expression of serum HMGB 1 in septic patients in a preliminary study (Figure 6). Although the relationship between HMGB1 and ATF3 is not completely understood, we showed that in ATF3 KO mice, the serum HMGB1 level was elevated; however, administering AAV-ATF3 vector could reverse the HMGB1 level. These data indicate that ATF3 exhibits a protective effect against endotoxemia through an anti-inflammatory mechanism involving HNGB1 molecule. Further, HMGB1 could be a useful marker for sepsis.

The role of TNF-*α* and IL-6 in inflammation has been firmly established. Both TNF-*α* and IL-6 are members of a group of cytokines that stimulate the acute phase reaction [32, 33]. ATF3 KO enhanced the LPS-induced increase of TNF-*α* and IL-6 levels in the lung tissue, and the enhancement persisted for at least 24 hrs for IL-6. These results imply that ATF3 negatively regulates IL-6 and TNF-*α* level which are in agreement with other endotoxemic studies [6]. Expression of IL-6 requires the activation of NF-*κ*B [34]. We found that ATF3 KO persistently increased the translocation of NF-*κ*B p65 (Figure 5(d)). Others have reported similar finding [35]. In addition, ATF3 siRNA was observed to increase the nuclear translocation NF-*κ*B p65 in macrophages (Figure 5(e)). Together, these data indicate that ATF3 decreases IL-6 presentation via NF-*κ*B signaling. Another inflammatory cytokine, nitric oxide (NO), is a major cause of septic shock [36]. We found that ATF3 deficiency increased serum NO concentration of endotoxemic mice which would aggravate septic syndrome, especially septic shock. 

Monocyte chemoattractant protein-1 (MCP-1) is a major chemoattractant for monocytes [37]. It plays an important role in the recruitment of monocytes or macrophages to the sites of injury and infection. Our data demonstrated that LPS induced a dramatic increase in MCP-1 production in the lung tissues followed by an increase in the recruitment of macrophages that exacerbated lung injury. Macrophages secrete TNF-*α*, and TNF-*α* elicits MCP-1 release; one can expect a positive feedback loop generated by these cytokines that will aggravate the inflammation of endotoxemia [38]. MCP-1 is involved in the pathogeneses of several diseases characterized by monocyte infiltration, such as psoriasis, rheumatoid arthritis, atherosclerosis and renal inflammation [37, 39]. However, the role of MCP1 in endotoxemia is not clear due to inadequate data. Our results showed that MCP1 played a pivotal role in endotoxemic lung injury, which was regulated negatively by ATF3. Furthermore, MCP-1 gene transcription is mediated by the transcription factor NF-*κ*B [5], and there are two NF-*κ*B binding sites on MCP-1 gene suggesting that ATF3 most likely inhibits MCP-1 expression by decreasing NF-*κ*B signaling, which was substantiated by decreased NF-*κ*B p65 translocation seen in our study (Figures 5(d) and 5(e)).

HMGB1 is a ligand for TLR 4 [11], and TLR 4 signaling is a common pathway for immune recognition of microbial invasion. Once the activation of immune system is elicited, activated macrophages produce HMGB1 and other inflammation proteins [18] leading to more HMGB1 expression, and as inflammation progresses, it will lead to multiple organs failure. That is a vicious circle; if can be blocked, septic mortality may be prevented. Here we have shown that HMGB1 was secreted to the cytoplasm of alveolar cell and serum after LPS treatment in mice. As soon as HMGB 1 enters blood, it would affect systemic organs. HMGB1 secretion in ATF3 KO mice was more than that of control mice after LPS treatment (Figure 8(a)) and reversed to control level after AAV-ATF3 transfection in KO mice. Similar observations were found in endotoxemic lung tissue and LPS-treated macrophages, confirming that ATF3 deficiency would increase HMGB1 cytosolic presentation (Figures 8 and 9(c)). However, we don't prove what ATF3 altered is transcription of HMGB1 gene or nuclear-to-cytosolic translocation of HMGB1. Certainly, it is unable to distinguish this issue up from our study. In theory, these two cases are likely to occur. Of course, if we can recognize whether knockout of ATF3 alters transcription of HMGB1 gene, not only enhancing nuclear-to-cytosolic translocation, it will be better to regulate HMGB1 in inflammatory process. Late study indicated that after direct nerve injury there was an increase in cytoplasmic HMGB1 protein expression and a decrease in nuclear HMGB1 protein expression. However, total HMGB1 protein content in the dorsal root ganglia was not altered by nerve injury [40]. Whether the same situation will happen in sepsis model, we will check that in our future studies.

In LPS-induced mouse endotoxemia model, the survival rate was lower in *atf3* ^−/−^ group compared with control group but returned to near control level after transfection of AAV-ATF3 in *atf3* ^−/−^ mice. The recovery of the survival rate also is accompanied with the reversal of NO level and lung injury (Figures 7(a)–7(d)). The *in vivo* ATF3 KO results also were collaborated by the *in vitro* macrophages treated with ATF3 siRNA.

Activation of the innate immune system is an essential step in raising an antimicrobial response to pathogens; however, during sepsis, some people die early from sepsis-induced hyperinflammation, an uncontrolled over activation of the innate immune system. Stimulation of macrophages by LPS produces NO which can facilitate the release of HMGB1 [41], and our data revealed that NO was increased in ATF3-null mice (Figure 5(b)). We can reasonably assume that ATF3 inhibits HMGB1 by decreasing NO production. Furthermore, HMGB1 signaling requires activation of NF-*κ*B [42], and HMGB-1 signaling helps the activation of the (NF-*κ*B) [11]. Thus, mutual activation exists between HMGB 1 and NF-*κ*B. In our study, regardless of *in vivo* or* in vitro* experiments, *atf3* deficiency increased the presentation of HMGB1 and NF-*κ*B. However, in ATF3 KO mice, LPS-induced elevation of HMGB1 appeared to precede that of NF-*κ*B (Figures 5(d) and 8(a)). These data imply that ATF3 is required for the protection of endotoxemia through inhibiting HMGB1 secretion and release. Nevertheless, more studies are needed to delineate the mechanisms of the modulation HMGB1 which may allow us to have a better control of the inflammatory processes.

The neutralization of inflammatory mediators with endotoxin-specific antibodies [43], tumor necrosis factor (TNF) inhibitors [44], or interleukin receptor antagonists [45] does not improve the overall survival of people with sepsis. In spite of the current advances in antibiotic therapy and intensive care, sepsis is still the most common reason of death in intensive care units [46]. In this study, we demonstrated that ATF3 is involved in most steps of innate immune response after pathogen invasion; ATF3 affects neutrophils recruitment, macrophage activation, proinflammatory cytokines production, and HMGB1-TLR4 circle. ATF3 presents in the early stage (3 hrs, Figure 1) of endotoxemia to prevent macrophage activation and inhibit NO, IL-6, and TNF-*α* release that eventually will trigger an uncontrolled systemic inflammatory response that may lead to sepsis. ATF3 can block the processes of innate immune response induced by invading pathogens in the early stage but also regulate the late-acting mediators of sepsis, such as HMGB1. Therefore, suppressing LPS-induced inflammation with ATF3 induction will be an important target in pharmacologic treatment of sepsis.

## 5. Conclusions

ATF3 is a negative regulator of HMGB1 and other pro-inflammatory cytokines and chemokines. ATF3 participates in multiple stages of septic syndrome, including early and late stages. Inhibition of inflammation and blockade of HMGB1-TLR 4 vicious circle by ATF3 during endotoxemia may prevent patients from sepsis-mediated mortality.

## Figures and Tables

**Table 1:** Primers used.

Target gene	Sense primer	Antisense primer
TNF-*α*	CCCTCACACTCAGATCATCTTCT	GCTACGACGTGGGCTACAG
IL-6	TAGTCCTTCCTACCCCAATTTCC	TTGGTCCTTAGCCACTCCTTC
Actin	GGCTGTATTCCCCTCCATCG	CCAGTTGGTAACAATGCCATGT

**Table 2:** Patient demographics for sepsis (–) and sepsis (+) patients.

Patient characteristics	Septic patients (*n* = 10)	Nonseptic inpatients (*n* = 10)	Healthy volunteers (*n* = 4)
Age in years (range)	67.7 (35–85)	51.6 (35–69)	35.23 (25–43)
Sex	70% men, 30% women	50% men, 50% women	100% women
Race	100% Asian	100% Asian	100% Asian
Comorbidities*	10	7	0
Admission diagnosis			
Sepsis	1	0	
Pneumonia	4	0	0
Urinary tract infection	4	0	0
Cellulitis	1	0	0
Non infection^§^	0	10	0

*Including diabetes, hypertension, stroke, Malignancy, and liver cirrhosis.

^§^Including fracture, lumbago, urolithiasis, myocardium infarction, hyponatremia, stroke, hepaticencephalopathy, carbon monoxide poisoning, and uterine myoma.
